# Congenital Occlusion and Dilation of Lymph Channels

**Published:** 1878-08

**Authors:** 


*Congenital Occlusion and Dilatation of Lymph Channels.* By SAMUEL C. BUSEY, M. D., Professor of the Theory and Practice of Medicine in Medical Department of the University of Georgetown, etc. New York: Wm. Wood & Co. 800. pp. 187.

A series of eighty-eight cases of diseases affecting lymph channels are here presented, the majority of which have been obtained from foreign sources. Considerable labor must have been involved in collecting these cases, and in preparing this monograph. The subject is one upon which information is desirable as little has been known concerning it. The volume is for the most part a re-production of what has recently appeared, in serial form, in the American Journal of Obstetrics. Numerous wood cut illustrations accompany the translated reports of cases and are quite an aid to the reader. The statements of the author are instructive and indicative of the wide re-search he has made.

